# Trends in opioid utilization in Hungary, 2006–2020: A nationwide retrospective study with multiple metrics

**DOI:** 10.1002/ejp.2011

**Published:** 2022-08-12

**Authors:** Zsófia Engi, Ria Benkő, Gyöngyvér Soós, Délia Szok, Melinda Csenki, Emese Csüllög, Attila Balog, Dezső Csupor, Réka Viola, Péter Doró, Mária Matuz

**Affiliations:** ^1^ Institute of Clinical Pharmacy, Faculty of Pharmacy University of Szeged Szeged Hungary; ^2^ Central Pharmacy Department, Albert Szent‐Györgyi Health Center University of Szeged Szeged Hungary; ^3^ Emergency Department, Albert Szent‐Györgyi Health Center University of Szeged Szeged Hungary; ^4^ Department of Neurology, Albert Szent‐Györgyi Health Center University of Szeged Szeged Hungary; ^5^ Department of Oncotherapy, Albert Szent‐Györgyi Health Center University of Szeged Szeged Hungary; ^6^ Department of Anesthesiology and Intensive Care, Albert Szent‐Györgyi Health Center University of Szeged Szeged Hungary; ^7^ Department of Rheumatology and Immunology, Albert Szent‐Györgyi Health Center University of Szeged Szeged Hungary; ^8^ Institute for Translational Medicine, Medical School University of Pécs Pécs Hungary

## Abstract

**Background:**

Opioid use is well documented in several countries: some countries struggle with overuse, whereas others have almost no access to opioids. For Europe, limited data are available. This study analysed Hungarian opioid utilization in ambulatory care between 2006 and 2020.

**Methods:**

We obtained national drug utilization data on reimbursed opioid analgesics (ATC code: N02A) from a national health insurance database for a 15‐year period. We investigated utilization trends, using three volume‐based metrics [defined daily dose per 1000 inhabitants per day (DID), oral morphine equivalent per 1000 inhabitants per day, packages dispensed per 1000 inhabitants per year]. We stratified data based on administration routes, analgesic potency and reimbursement categories.

**Results:**

Total opioid utilization increased during the study period according to all three metrics (74% in DID) and reached 5.31 DID by 2020. Upward trends were driven by an increase both in weak and strong opioid use (79% vs. 53%). The most commonly used opioids were fentanyl (in the strong category; 0.76 DID in 2020) and tramadol (in the weak category; 2.62 DID in 2020). Overall, tramadol was also the most commonly used opioid throughout the study period. Oral administration of opioid medications was dominant. Based on reimbursement categories, musculoskeletal pain was becoming a more frequent indication for opioid use (1552% increase in DID), while opioid use for cancer pain declined significantly during the study period (−33% in DID).

**Conclusions:**

Our low utilization numbers might indicate underuse of opioid analgesia, especially for cancer pain.

**Significance:**

This study was one of the recent opioid utilization studies using three volume‐based metrics, covering a long time period. To our knowledge, this was also the first national, population level study describing opioid utilization in Hungary. National opioid utilization data suggested not an overuse but rather an underuse of opioid analgesics in a developed, Central European country.

## 1 INTRODUCTION

Opioids have been used as analgesics since ancient times, but pure opioids were introduced in therapy only at the beginning of the 19th century (Sabatowski et al., 2004). Opioid analgesics are remarkably effective in relieving moderate–severe acute or chronic pain, and no equivalent alternatives have been discovered yet. Weak and strong opioids are recommended as second or third‐line drugs in different types of primary and secondary chronic pain syndromes, and also in peripheral or central neuropathic pain (Attal et al., 2010; Dowell et al., 2016; Finnerup et al., 2015).

Pain is one of the most common reasons a patient visits a doctor. Surveys in different European countries estimated up to 30% of the participants reporting chronic pain (Breivik et al., 2013; Häuser et al., 2015). Pain relief is a basic human right, and inadequate analgesia might lead to a decline in quality of life as it negatively impacts a person's physical, mental and social status (Vranken et al., 2018). However, there is a high risk of opioid abuse that has generated strict national and international regulations in order to optimize medical use and prevent illegal activities.

Opioid utilization varies wildly across the world. There is a well‐documented overuse crisis in the United States, and during the last decade, utilization has increased significantly in other developed countries as well (Bosetti et al., 2019; Karanges et al., 2018; Svendsen et al., 2011). However, fear of addiction and abuse as well as strict legislations (administration burdens) may contribute to hesitancy in prescribing opioids. Moreover, in many developing countries, opioids are immensely underused, and a large part of the world population (over 75%) has almost no access to opioid analgesics (Alqueres et al., 2015; Vranken et al., 2018).

According to the 2010 report of the International Narcotics Control Board (INCB), given the average consumption of narcotic drugs, Hungary ranks 19th out of 42 in the Europe region and 25th out of 179 countries in the world (INCB Special Report Covering 2007–2009, 2010). Based on the data provided by the INCB, a recent comprehensive study analysing the trends in opioid consumption in 22 selected European countries (including Hungary) was published in 2019 (Bosetti et al., 2019) and its data were reused in a distinct paper (Häuser, Buchser, et al., 2021). However, as Bäckryd et al. (2021) pointed out, due to the questionable methodology, INCB reports might overestimate opioid consumption. Besides the above‐mentioned report and manuscripts, both with suboptimal methodology, no other data have been published for Hungary. Published opioid utilization data (in the scientific literature) are scarce from other countries as well. Aside from an Australian (Karanges et al., 2018), a French (Hider‐Mlynarz et al., 2018), a Danish (Nissen et al., 2019), a German (Rosner et al., 2019) and an Irish study (Norris et al., 2021), no thorough national opioid utilization data were published in the last few years.

The aim of this study was to gain comprehensive knowledge on the Hungarian national opioid utilization trends in ambulatory care over a 15‐year period from 2006 to 2020.

## 2 METHODS

### 2.1 Setting and data sources

In Hungary, a single health insurance fund (the National Health Insurance Fund of Hungary, Hungarian acronym: NEAK) provides health coverage for all the population (almost 10 million people).

Raw (package level) national drug utilization data on reimbursed opioid analgesics (ATC code: N02A) were obtained from the NEAK database for a 15‐year period: 2006–2020. The database contains the following aggregated data on each reimbursed ambulatory care prescription dispensed at community pharmacies in Hungary: drug name; drug strength; package size; Anatomical Therapeutic Chemical Classification (ATC) code; active ingredient; reimbursement category; number of dispensed and reimbursed packages; total retail cost; and total reimbursement cost.

With the exception of some non‐reimbursed tramadol and tramadol combination products, the NEAK database provides a nearly 100% drug coverage for opioid dispensing.

Ambulatory care prescriptions include prescriptions for nursing home patients and prescriptions issued by private healthcare facilities (including dentists). However, non‐reimbursed drugs and opioids given to hospital inpatients are not registered in this database. All opioid products are prescription‐only medicines but reimbursement varies: depending on the active ingredient, the strength and the indication, opioid drugs can be reimbursed at a rate of 25% (general), 90% (high) or 100% (accentuated). The high reimbursement category is linked only to musculoskeletal indications, and only oxycodone and fentanyl products of a certain strength can be prescribed this way. Accentuated reimbursement is linked only to oncological indications, whereas the general category has no linked indication and almost all active ingredients can be prescribed with these reimbursements (Table S1).

### 2.2 Analysis

We analysed annual opioid utilization from 2006 to 2020 using three volume‐based metrics [DDD (defined daily dose) per 1000 inhabitants per day, OME (oral morphine equivalent) per 1000 inhabitants per day and packages dispensed per 1000 inhabitants per year]. All metrics were standardized by the annual number of the total Hungarian population, collected from the Eurostat database (Database ‐ Eurostat, n.d.).

We calculated DDD per 1000 inhabitants per day by using the World Health Organization's (WHO) ATC/DDD system (2020 version). We focused our analysis on the N02A ATC subgroup. For combination products we calculated DDD per 1000 inhabitants per day using the DDDs expressed in unit dose (UD) as found in WHO's ‘List of DDDs combined products’ (WHOCC – List of DDDs Combined Products, n.d.).

DDD per 1000 inhabitants per day was converted into OME per 1000 inhabitants per day, using the methodology of Karanges et al. (2018) for the calculations (Figure S1). OME conversion factors were derived from a number of the existing literature (Karanges et al., 2018; Nielsen et al., 2016; Nissen et al., 2019), since we found no adequately comprehensive paper that covered all the active substances and administration routes analysed in our study (Table S2). In case of combination products, only the core opioid component was taken into account when calculating OME per 1000 inhabitants per day based on the study by Nielsen et al.

The basis of OME is the concept that different doses of opioids of varying potency might have an identical analgesic effect (Nielsen et al., 2016). Because it takes the analgesic potency of each opioid into account (Karanges et al., 2018) and eliminates the assumptions generated by the division of the quantity of a drug by a defined dose (Nielsen et al., 2017), it appears to provide a more fitting comparison of opioid use (Nissen et al., 2019). Therefore OME per 1000 inhabitants per day might be a more clinically relevant utilization metric than DDD per 1000 inhabitants per day (Karanges et al., 2018).

We stratified our data based on administration routes, analgesic potency and reimbursement categories.

We considered the following administration routes: oral, transdermal, rectal and parenteral. Sublingual formulations were not taken into account as they were on the Hungarian drug market only for a short period of time, at a relatively high cost.

To maintain the proper systemic drug levels and sustain the ideal release rate of the drug throughout use, transdermal patches usually contain an excess of the drug substance. As this residual amount will not be used by the patient, it was not taken into consideration during our analysis. For buprenorphine and fentanyl transdermal patches, the actual amount of used opioid was calculated based on strength per hour and the intended duration of use (72 h).

We classified the opioids into weak and strong potency categories according to the WHO's three‐step analgesic ladder (Anekar & Cascella, 2021; Hider‐Mlynarz et al., 2018).

The category of weak opioids contained codeine combinations excl. Psycholeptics (N02AA59), codeine and paracetamol combinations (N02AJ06), dihydrocodeine (N02AA08), tramadol (N02AX02), and tramadol and paracetamol combination products (N02AJ13). There is no codeine only product on the Hungarian market.

The category of strong opioids encompassed morphine (N02AA01), hydromorphone (N02AA03), oxycodone (N02AA05), oxycodone and naloxone combination (N02AA55), pethidine (N02AB02), fentanyl (N02AB03), buprenorphine (N02AE01) and nalbuphine (N02AF02).

Reimbursement categories were studied because they can be linked to indications, which gives us an extensive insight into opioid use. Fentanyl patches are available in 12, 25, 50, 75 and 100 μg/h formulations. Reimbursement of 50, 75 and 100 μg/h patches is strictly linked to oncological indications (100% reimbursement, accentuated category), while 12 and 25 μg/h patches can also be prescribed for musculoskeletal pain (90% reimbursement, high reimbursement category). Similarly, 40 and 80 mg oxycodone oral formulations are linked to oncological indications (100% reimbursement, accentuated category), whereas 5, 10 and 20 mg products can also be prescribed for musculoskeletal pain (90% reimbursement, high reimbursement category).

Data analysis was performed for each year of the study period using the measures described above. Analyses were performed using R version 4.1.0 and Microsoft Excel.

## 3 RESULTS

### 3.1 Scale and trends of opioid utilization from 2006 to 2020

In 2020, total opioid utilization was 5.3 DDD per 1000 inhabitants per day, 275.1 OME per 1000 inhabitants per day and 278.3 packages per 1000 inhabitants per year. Absolute and relative use of different opioids are summarized in Tables 1, 2, 3, 4, 5, 6, respectively.

**TABLE 1:** Hungarian opioid use in the ambulatory care sector, expressed as DDD/1000 inhabitants/day (a: <0.01)

	2006	2007	2008	2009	2010	2011	2012	2013	2014	2015	2016	2017	2018	2019	2020	% change
**Total N02A**	3.05	3.15	3.37	3.50	3.60	3.79	3.85	4.01	4.07	4.16	4.25	4.64	4.99	5.09	5.31	74.30
**Administration route**																
Oral	2.45	2.50	2.70	2.81	2.88	3.04	3.09	3.21	3.24	3.33	3.39	3.79	4.14	4.25	4.48	82.90
Transdermal	0.48	0.55	0.58	0.60	0.65	0.67	0.68	0.72	0.76	0.76	0.79	0.79	0.79	0.79	0.77	62.05
Rectal	0.10	0.08	0.07	0.07	0.06	0.06	0.06	0.06	0.06	0.06	0.06	0.05	0.05	0.05	0.05	−51.97
Parenteral	0.02	0.02	0.02	0.02	0.02	0.01	0.02	0.01	0.01	0.01	0.01	0.01	0.01	0.01	0.01	−67.75
**Potency**																
**Strong opioids**	0.56	0.63	0.68	0.72	0.76	0.79	0.79	0.82	0.86	0.86	0.89	0.89	0.89	0.88	0.86	52.95
Morphine (N02AA01)	0.06	0.05	0.03	0.02	0.02	0.02	0.01	0.01	0.01	0.01	0.01	0.01	0.01	0.01	0.01	−82.05
Hydromorphone (N02AA03)	0.02	0.02	0.05	0.07	0.08	0.08	0.07	0.05	0.05	0.03	0.03	0.02	0.02	0.02	0.01	−30.40
Oxycodone (N02AA05)	a	0.01	0.02	0.02	0.01	0.02	0.02	0.03	0.04	0.05	0.06	0.06	0.07	0.06	0.06	355.56
Oxycodone and naloxone (N02AA55)	–	–	–	–	–	–	–	–	–	–	–	–	–	–	a	–
Pethidine (N02AB02)	a	a	a	a	a	a	a	a	a	a	–	–	–	–	–	–
Fentanyl (N02AB03)	0.48	0.54	0.55	0.57	0.62	0.66	0.68	0.72	0.75	0.76	0.79	0.79	0.79	0.78	0.76	59.90
Buprenorphine (N02AE01)	a	0.01	0.04	0.04	0.02	0.01	a	a	0.01	a	a	a	a	0.01	0.01	−11.16
Nalbuphine (N02AF02)	a	a	a	–	–	–	–	–	–	–	–	–	–	–	–	a
**Weak opioids**	2.49	2.51	2.69	2.78	2.84	3.00	3.06	3.19	3.21	3.30	3.36	3.75	4.10	4.22	4.45	79.10
Dihydrocodeine (N02AA08)	0.07	0.06	0.05	0.03	0.02	0.02	0.02	0.02	0.02	0.02	0.02	0.02	0.02	0.02	0.02	−74.50
Other codeine comb. (N02AA59)	0.08	0.07	0.07	0.06	0.06	0.06	0.06	0.06	0.06	0.05	0.05	0.05	0.05	0.05	0.05	−45.63
Codeine and paracetamol (N02AJ06)	a	a	–	–	–	–	–	–	–	–	–	–	–	–	–	–
Tramadol and paracetamol (N02AJ13)	–	–	–	–	a	–	a	–	a	a	–	0.54	1.12	1.52	1.76	–
Tramadol (N02AX02)	2.33	2.38	2.57	2.68	2.76	2.92	2.99	3.12	3.14	3.23	3.29	3.14	2.91	2.63	2.62	12.56
**Reimbursement category**																
General	1.82	1.93	2.15	2.31	2.40	2.57	2.67	2.80	2.84	2.95	3.02	3.43	3.80	3.95	4.18	130.30
High (musculoskeletal indication)	0.02	0.05	0.08	0.10	0.12	0.16	0.19	0.21	0.24	0.27	0.29	0.31	0.31	0.32	0.32	1551.83
Accentuated (Oncological indication)	1.21	1.16	1.14	1.09	1.07	1.06	0.99	0.99	0.98	0.94	0.93	0.90	0.88	0.83	0.81	−33.21
Other	a	a	a	a	a	a	a	a	a	a	a	a	a	a	a	12.97

**TABLE 2:** Hungarian opioid use in the ambulatory care sector, expressed as OME/1000 inhabitants/day (a: <0.01)

	2006	2007	2008	2009	2010	2011	2012	2013	2014	2015	2016	2017	2018	2019	2020	% change
**Total**	150.25	153.73	166.96	174.93	179.52	189.56	192.24	199.51	200.98	206.81	210.71	233.78	255.33	261.62	275.14	83.12
**Administration route**																
Oral	146.95	150.51	163.81	171.77	176.18	186.31	188.87	196.13	197.57	203.41	207.29	230.79	252.17	258.42	272.06	85.14
Transdermal	1.55	1.77	1.87	1.94	2.08	2.17	2.21	2.33	2.46	2.47	2.56	2.56	2.55	2.55	2.50	61.65
Rectal	a	a	a	a	a	a	a	a	a	a	a	a	a	a	a	–
Parenteral	1.75	1.45	1.29	1.22	1.26	1.08	1.17	1.05	0.96	0.93	0.85	0.43	0.61	0.65	0.58	−66.90
**Potency**																
**Strong opioids**	9.81	10.48	12.14	13.63	13.62	14.36	12.95	12.21	12.52	13.07	13.40	12.95	13.08	12.25	11.53	17.59
Morphine (N02AA01)	6.20	4.91	3.32	2.39	2.24	2.21	1.41	1.44	1.42	1.27	1.37	1.39	1.23	1.10	1.09	−82.42
Hydromorphone (N02AA03)	1.88	2.31	4.59	7.45	7.61	8.16	7.12	5.46	4.63	3.37	2.55	2.06	1.93	1.65	1.31	−30.40
Oxycodone (N02AA05)	0.13	1.45	2.32	1.82	1.67	1.80	2.18	2.95	3.99	5.95	6.93	6.93	7.36	6.96	6.62	5151.78
Oxycodone and naloxone (N02AA55)	–	–	–	–	–	–	–	–	–	–	–	–	–	–	0.01	–
Pethidine (N02AB02)	0.06	0.04	0.04	0.03	0.02	0.02	0.02	0.03	0.03	a	–	–	–	–	–	−100.00
Fentanyl (N02AB03)	1.55	1.74	1.77	1.84	2.02	2.13	2.20	2.32	2.44	2.46	2.55	2.56	2.55	2.52	2.47	59.90
Buprenorphine (N02AE01)	a	0.03	0.10	0.09	0.06	0.03	0.01	0.01	0.02	0.01	0.01	a	a	0.02	0.03	4597.08
Nalbuphine (N02AF02)	a	a	a	–	–	–	–	–	–	–	–	–	–	–	–	−100.00
**Weak opioids**	140.44	143.26	154.82	161.30	165.90	175.19	179.30	187.30	188.46	193.74	197.31	220.83	242.26	249.37	263.61	87.70
Dihydrocodeine (N02AA08)	1.04	0.91	0.75	0.45	0.31	0.26	0.26	0.26	0.28	0.32	0.31	0.30	0.29	0.29	0.26	−74.50
Other codeine comb. (N02AA59)	a	a	a	a	a	a	a	a	a	a	a	a	a	a	a	–
Codeine and paracetamol (N02AJ06)	0.01	a	–	–	–	–	–	–	–	–	–	–	–	–	–	−100.00
Tramadol and paracetamol (N02AJ13)	–	–	–	–	a	–	a	–	a	a	–	32.27	67.46	91.44	105.85	–
Tramadol (N02AX02)	139.39	142.34	154.07	160.85	165.60	174.94	179.04	187.04	188.18	193.42	196.99	188.26	174.51	157.64	157.49	12.98
**Reimbursement category**																
General	102.72	110.50	124.24	134.33	140.42	150.07	156.23	164.47	166.61	173.25	177.84	202.09	224.65	233.86	247.94	141.36
High (musculosceletal indication)	0.06	0.56	1.22	1.20	1.20	1.58	1.93	2.21	2.68	3.16	3.57	3.72	3.73	3.73	3.79	5981.30
Accentuated (Oncological indication)	47.24	42.46	41.30	39.16	37.66	37.69	33.89	32.62	31.45	30.14	29.03	27.71	26.69	23.80	23.17	−50.96
Other	0.22	0.22	0.20	0.24	0.24	0.22	0.19	0.22	0.24	0.26	0.26	0.26	0.26	0.24	0.24	8.12

**TABLE 3:** Hungarian opioid use in the ambulatory care sector, expressed as packages/1000 inhabitants/year (a: <0.01)

	2006	2007	2008	2009	2010	2011	2012	2013	2014	2015	2016	2017	2018	2019	2020	% change
**Total**	214.75	205.15	214.08	215.80	218.08	227.75	229.93	238.59	250.74	254.28	257.04	265.54	276.49	273.97	278.34	29.61
**Administration route**																
Oral	177.73	170.42	179.42	182.01	183.16	191.51	193.54	201.25	212.00	214.99	216.49	226.87	238.47	235.99	240.80	35.48
Transdermal	9.53	11.93	13.54	14.54	16.15	17.48	18.30	19.82	21.73	22.54	23.84	24.48	24.75	24.94	24.88	160.94
Rectal	22.98	19.36	17.70	16.16	15.65	15.59	14.92	14.46	14.32	14.10	14.04	13.21	12.14	11.39	11.17	−51.41
Parenteral	4.50	3.43	3.42	3.09	3.12	3.16	3.17	3.06	2.69	2.65	2.67	0.97	1.13	1.65	1.50	−66.61
**Potency**																
**Strong opioids**	12.03	14.52	16.74	17.99	19.60	20.98	21.54	22.99	25.17	26.39	28.12	28.88	29.27	29.16	28.98	140.79
Morphine (N02AA01)	2.07	1.62	1.13	0.85	0.83	0.79	0.61	0.60	0.58	0.55	0.62	0.83	0.98	0.82	0.82	−60.43
Hydromorphone (N02AA03)	0.25	0.29	0.89	1.70	1.82	1.85	1.59	1.20	1.02	0.77	0.63	0.50	0.42	0.37	0.29	15.68
Oxycodone (N02AA05)	0.07	0.62	1.11	0.85	0.76	0.82	1.01	1.32	1.79	2.53	3.04	3.07	3.13	3.04	2.99	4097.49
Oxycodone and naloxone (N02AA55)	–	–	–	–	–	–	–	–	–	–	–	–	–	–	0.01	–
Pethidine (N02AB02)	0.10	0.06	0.08	0.05	0.04	0.03	0.04	0.05	0.05	0.01	–	–	–	–	–	−100.00
Fentanyl (N02AB03)	9.53	11.69	12.87	13.88	15.74	17.26	18.24	19.75	21.61	22.48	23.78	24.46	24.74	24.77	24.66	158.74
Buprenorphine (N02AE01)	a	0.24	0.67	0.66	0.41	0.23	0.06	0.07	0.12	0.06	0.06	0.02	0.01	0.17	0.22	4844.45
Nalbuphine (N02AF02)	a	a	a	–	–	–	–	–	–	–	–	–	–	–	–	−100.00
**Weak opioids**	202.71	190.62	197.33	197.81	198.47	206.77	208.39	215.60	225.57	227.89	228.91	236.66	247.22	244.81	249.36	23.01
Dihydrocodeine (N02AA08)	3.12	2.54	1.88	1.33	1.32	1.24	1.22	1.03	1.20	1.49	1.53	1.57	1.50	1.60	1.56	−49.96
Other codeine comb. (N02AA59)	20.54	17.48	16.14	14.86	14.45	14.39	13.85	13.56	13.42	13.26	13.21	12.47	11.81	11.38	11.17	−45.63
Codeine and paracetamol (N02AJ06)	0.04	a	–	–	–	–	–	–	–	–	–	–	–	–	–	−100.00
Tramadol and paracetamol (N02AJ13)	–	–	–	–	a	–	a	–	a	a	–	20.49	48.77	66.14	77.61	–
Tramadol (N02AX02)	179.02	170.60	179.31	181.62	182.70	191.15	193.32	201.00	210.95	213.13	214.18	202.13	185.14	165.69	159.03	−11.17
**Reimbursement category**																
General	159.60	157.88	168.50	173.11	175.73	184.69	188.69	196.51	207.17	210.65	212.50	221.20	232.79	231.83	236.90	48.43
High (musculosceletal indication)	0.78	2.65	4.35	5.06	6.17	7.78	9.13	10.50	12.21	13.50	14.71	15.59	15.74	16.01	16.13	1960.60
Accentuated (Oncological indication)	54.08	44.36	40.98	37.36	35.92	35.03	31.87	31.29	31.06	29.82	29.54	28.49	27.70	25.89	25.08	−53.63
Other	0.28	0.26	0.24	0.27	0.26	0.25	0.25	0.28	0.30	0.31	0.29	0.26	0.26	0.24	0.23	−18.09

**TABLE 4:** Hungarian opioid use in the ambulatory care sector, expressed as DDD/1000 inhabitants/day in percentage

	2006	2007	2008	2009	2010	2011	2012	2013	2014	2015	2016	2017	2018	2019	2020
**Total N02A**	3.05 (100%)	3.15 (100%)	3.37 (100%)	3.50 (100%)	3.60 (100%)	3.79 (100%)	3.85 (100%)	4.01 (100%)	4.07 (100%)	4.16 (100%)	4.25 (100%)	4.64 (100%)	4.99 (100%)	5.09 (100%)	5.31 (100%)
**Administration route**															
Oral	80.46%	79.43%	80.06%	80.34%	79.81%	80.20%	80.29%	80.18%	79.56%	79.99%	79.77%	81.65%	83.04%	83.47%	84.43%
Transdermal	15.67%	17.43%	17.27%	17.29%	17.94%	17.72%	17.71%	17.98%	18.68%	18.32%	18.61%	17.06%	15.81%	15.45%	14.57%
Rectal	3.14%	2.56%	2.18%	1.92%	1.80%	1.71%	1.61%	1.49%	1.46%	1.40%	1.37%	1.18%	1.00%	0.92%	0.86%
Parenteral	0.73%	0.58%	0.49%	0.45%	0.46%	0.37%	0.39%	0.34%	0.30%	0.29%	0.26%	0.11%	0.14%	0.16%	0.14%
**Potency**															
Strong opioids	18.39%	20.16%	20.25%	20.59%	21.10%	20.90%	20.44%	20.37%	21.05%	20.71%	20.98%	19.14%	17.76%	17.21%	16.14%
Weak opioids	81.61%	79.84%	79.75%	79.41%	78.90%	79.10%	79.56%	79.63%	78.95%	79.29%	79.02%	80.86%	82.24%	82.79%	83.86%
**Reimbursement category**															
General	59.62%	61.36%	63.72%	65.94%	66.73%	67.75%	69.28%	69.86%	69.71%	70.78%	71.09%	73.85%	76.13%	77.49%	78.77%
High	0.63%	1.68%	2.51%	2.89%	3.46%	4.16%	4.84%	5.28%	6.00%	6.46%	6.88%	6.65%	6.22%	6.20%	5.96%
Accentuated	39.63%	36.84%	33.66%	31.05%	29.70%	27.99%	25.79%	24.74%	24.18%	22.64%	21.92%	19.39%	17.56%	16.22%	15.19%
Other	0.13%	0.12%	0.11%	0.12%	0.12%	0.10%	0.09%	0.12%	0.11%	0.12%	0.11%	0.10%	0.09%	0.09%	0.08%

**TABLE 5:** Hungarian opioid use in the ambulatory care sector, expressed as OME/1000 inhabitants/day in percentage

	2006	2007	2008	2009	2010	2011	2012	2013	2014	2015	2016	2017	2018	2019	2020
**Total**	150.25 (100%)	153.73 (100%)	166.96 (100%)	174.93 (100%)	179.52 (100%)	189.56 (100%)	192.24 (100%)	199.51 (100%)	200.98 (100%)	206.81 (100%)	210.71 (100%)	233.78 (100%)	255.33 (100%)	261.62 (100%)	275.14 (100%)
**Administration route**															
Oral	97.80%	97.91%	98.11%	98.19%	98.14%	98.29%	98.25%	98.30%	98.30%	98.36%	98.38%	98.72%	98.76%	98.78%	98.88%
Transdermal	1.03%	1.15%	1.12%	1.11%	1.16%	1.14%	1.15%	1.17%	1.22%	1.19%	1.22%	1.10%	1.00%	0.97%	0.91%
Rectal	0.00%	0.00%	0.00%	0.00%	0.00%	0.00%	0.00%	0.00%	0.00%	0.00%	0.00%	0.00%	0.00%	0.00%	0.00%
Parenteral	1.17%	0.94%	0.77%	0.70%	0.70%	0.57%	0.61%	0.53%	0.48%	0.45%	0.41%	0.18%	0.24%	0.25%	0.21%
**Potency**															
Strong opioids	6.53%	6.82%	7.27%	7.79%	7.59%	7.58%	6.73%	6.12%	6.23%	6.32%	6.36%	5.54%	5.12%	4.68%	4.19%
Weak opioids	93.47%	93.18%	92.73%	92.21%	92.41%	92.42%	93.27%	93.88%	93.77%	93.68%	93.64%	94.46%	94.88%	95.32%	95.81%
**Reimbursement category**															
General	68.37%	71.88%	74.41%	76.79%	78.22%	79.17%	81.27%	82.43%	82.90%	83.77%	84.40%	86.44%	87.98%	89.39%	90.11%
High	0.04%	0.36%	0.73%	0.69%	0.67%	0.83%	1.00%	1.11%	1.33%	1.53%	1.70%	1.59%	1.46%	1.42%	1.38%
Accentuated	31.44%	27.62%	24.73%	22.38%	20.98%	19.88%	17.63%	16.35%	15.65%	14.57%	13.78%	11.85%	10.45%	9.10%	8.42%
Other	0.15%	0.14%	0.12%	0.13%	0.13%	0.11%	0.10%	0.11%	0.12%	0.13%	0.12%	0.11%	0.10%	0.09%	0.09%

**TABLE 6:** Hungarian opioid use in the ambulatory care sector, expressed as packages/1000 inhabitants/year in percentage

	2006	2007	2008	2009	2010	2011	2012	2013	2014	2015	2016	2017	2018	2019	2020
**Total**	214.75 (100%)	205.15 (100%)	214.08 (100%)	215.80 (100%)	218.08 (100%)	227.75 (100%)	229.93 (100%)	238.59 (100%)	250.74 (100%)	254.28 (100%)	257.04 (100%)	265.54 (100%)	276.49 (100%)	273.97 (100%)	278.34 (100%)
**Administration route**															
Oral	82.76%	83.07%	83.81%	84.34%	83.99%	84.09%	84.17%	84.35%	84.55%	84.55%	84.22%	85.44%	86.25%	86.14%	86.51%
Transdermal	4.44%	5.82%	6.32%	6.74%	7.41%	7.68%	7.96%	8.31%	8.67%	8.86%	9.27%	9.22%	8.95%	9.10%	8.94%
Rectal	10.70%	9.44%	8.27%	7.49%	7.18%	6.85%	6.49%	6.06%	5.71%	5.55%	5.46%	4.98%	4.39%	4.16%	4.01%
Parenteral	2.10%	1.67%	1.60%	1.43%	1.43%	1.39%	1.38%	1.28%	1.07%	1.04%	1.04%	0.37%	0.41%	0.60%	0.54%
**Potency**															
Strong opioids	5.60%	7.08%	7.82%	8.33%	8.99%	9.21%	9.37%	9.64%	10.04%	10.38%	10.94%	10.88%	10.59%	10.64%	10.41%
Weak opioids	94.40%	92.92%	92.18%	91.67%	91.01%	90.79%	90.63%	90.36%	89.96%	89.62%	89.06%	89.12%	89.41%	89.36%	89.59%
**Reimbursement category**															
General	74.32%	76.96%	78.71%	80.22%	80.58%	81.09%	82.06%	82.36%	82.62%	82.84%	82.67%	83.30%	84.19%	84.62%	85.11%
High	0.44%	1.56%	2.42%	2.78%	3.37%	4.06%	4.72%	5.22%	5.76%	6.28%	6.79%	6.87%	6.60%	6.79%	6.70%
Accentuated	567.27%	371.86%	302.65%	256.99%	222.40%	200.35%	174.15%	157.88%	142.94%	132.33%	123.90%	116.35%	111.96%	103.79%	100.81%
Other	1.23%	1.32%	1.38%	1.67%	1.66%	1.58%	1.65%	1.96%	2.11%	2.17%	2.09%	1.99%	2.11%	2.11%	2.07%

During the study period (2006–2020), total opioid utilization increased monotonically according to all three metrics (Figure S2). There was a 74.3% increase in DDD per 1000 inhabitants per day (Table 1), an 83.1% increase in OME per 1000 inhabitants per day (Table 2) and a 29.6% increase in packages per 1000 inhabitants per year (Table 3).

### 3.2 Pattern and trends of strong and weak opioid utilization

Upward utilization trends were driven by an increase both in weak and strong opioid use, although the increase in weak opioid utilization was generally more pronounced (Tables 1, 2, 3; Figure S3).

Utilization of weak opioids increased by 79.1% according to DDD per 1000 inhabitants per day, 87.7% according to OME per 1000 inhabitants per day and 23.0% according to packages per 1000 inhabitants per year. Utilization of strong opioids increased by 53.0%, 17.6% and 140.8% according to DDD per 1000 inhabitants per day, OME per 1000 inhabitants per day and packages per 1000 inhabitants per year, respectively.

In 2020, the utilization of strong opioids was 0.9 DDD per 1000 inhabitants per day, 11.5 OME per 1000 inhabitants per day and 29.0 packages per 1000 inhabitants per year while the utilization of weak opioids was 4.5 DDD per 1000 inhabitants per day, 263.6 OME per 1000 inhabitants per day and 249.4 packages per 1000 inhabitants per year. Thus, weak opioids accounted for the majority of total opioid use in 2020, covering more than 80% of utilization according to all three metrics. Strong opioids altogether represented approximately 4%, 10% and 16% of the overall opioid utilization in 2020 (in OME per 1000 inhabitants per day, in packages per 1000 inhabitants per year and in DDD per 1000 inhabitants per day, respectively). The most commonly used opioid was fentanyl in the strong category and tramadol in the weak category.

Overall, throughout the study period, the most commonly used opioid was tramadol (Tables 1, 2, 3). The increased use of tramadol and paracetamol combinations reduced the utilization of tramadol only products. Tramadol and tramadol combination products together steadily covered more than 75% of the opioid use during the study period according to all three metrics. Utilization of fentanyl and oxycodone products was also noteworthy, whereas other opioid drugs (codeine, dihydrocodeine, buprenorphine, hydromorphone, morphine) altogether amounted to <10% of opioid use according to all three metrics.

### 3.3 Pattern and trends of route of administration

Oral administration of opiod medications was dominant during the study period (more than 79% of the overall utilization according to all three metrics). There was also an increase in oral opioid analgesic use according to all three metrics; the highest increase was observed in the OME metric (above 85%) (Table 2).

Utilization of transdermal (fentanyl) patches also showed an increase during the study period (around 60% according to DDD and OME per 1000 inhabitants per day, and 160% according to packages per 1000 inhabitants per year) (Tables 1, 2, 3). By the end of the study period, transdermal formulations were the second most used according to all three metrics (Tables 4, 5, 6).

Parenteral opioid analgesics were rarely used in the Hungarian ambulatory care at the beginning of the study period and their use became even more scarce by the end of the study period (around 0.1%–0.5%). In rectal formulations, only a few active ingredients (codeine and tramadol) were available, and the utilization of this form was minor and showed a decreasing trend (Tables 1 and 3).

### 3.4 Patterns and trends in indication

There was a pronounced increase in the use of fentanyl patches and oxycodone products in the high reimbursement category, which indicates that musculoskeletal pain was becoming a more and more frequent indication for opioid drug use. There was also an increase in the general reimbursement category where no conclusion could be drawn regarding the therapeutic indications (indication linkage was not possible). However, according to all three metrics, opioid use for cancer pain (100% reimbursement) declined significantly during the study period (−33.2% in DDD per 1000 inhabitants per day, −50.9% in OME per 1000 inhabitants per day and −53.6% in packages per 1000 inhabitants per year) (Figure S5).

## 4 DISCUSSION AND CONCLUSIONS

To our knowledge, this was the first national study describing the characteristics of opioid utilization within ambulatory care settings in Hungary.

According to all three metrics, we found a clear increase in total opioid utilization between 2006 and 2020, which can be explained by several factors. There is a close correlation between the age distribution of the population and the prevalence of pain (Kalkman et al., 2019; Nissen et al., 2019) so possible explanations for the increase in opioid use are the aging of the population and longer lifespan: life expectancy increased by approximately 3 years during the study period (Statistics|Eurostat, n.d.). There was also a shift from hospital care to ambulatory care as the number of hospital beds was reduced in 2007 by approximately 10% (NEAK, 2020), so more patients with pain might be treated in ambulatory care.

The general increase in opioid use was consistent with observations from other countries. However, comparing our results was difficult because of methodical differences, lack of comparable numeric data and major differences in the duration, setting and timeline of studies, so comparisons and conclusions should be interpreted cautiously.

During our 15‐year study period, utilization of weak opioids increased by 88% and utilization of strong opioids increased by 18% in Hungary according to OME per 1000 inhabitants per day, while during a 19‐year study period (1999–2018), the same metric showed a 44% and a 33% increase in Demark, respectively (Nissen et al., 2019).

At the end of our study period, utilization of weak opioids was several‐fold higher in Hungary than utilization of strong opioids (according to all three metrics). This was in contrast with Australia, where there was a shift toward strong opioid utilization between 2006 and 2015 (Karanges et al., 2018).

Based on two of our metrics (DDD per 1000 inhabitants per day and packages per 1000 inhabitants per year), fentanyl was steadily the dominating strong opioid in Hungary during the study period. As transdermal fentanyl patches are easy to use even in case of dysphagia, this observation is reasonable. In a recent study, fentanyl was also found to be the most frequently used strong opioid in outpatient settings in Germany (Rosner et al., 2019).

Oxycodone, one of the main drivers of the opioid epidemic in the United States, represented a relatively small part of the overall opioid utilization in Hungary during the study period, although a significant increase could be detected. However, this was not nearly as major as in France, where oxycodone consumption increased considerably in total care, according to two separate studies conducted between 2006 and 2015 (Hider‐Mlynarz et al., 2018) and between 2004 and 2017 (Chenaf et al., 2019). As oxycodone is a suitable alternative when fentanyl patches cannot provide adequate pain relief or if cannot be tolerated, and a wide variety of strength is available for easy dose titration, further increase in use can be expected.

Among weak opioids, tramadol and tramadol combinations were the most used in Hungary, also covering most of the overall opioid utilization. This mirrors the upward trends of tramadol use in several other countries, such as Scotland (Ruscitto et al., 2015), France (Hider‐Mlynarz et al., 2018), Denmark (Nissen et al., 2019), Norway (Muller et al., 2019), Spain (Hurtado et al., 2020), Ireland (Norris et al., 2021) and Taiwan (Chen et al., 2021).

Long‐term use of tramadol is currently debatable as there is moderate evidence to support its benefit (Petzke et al., 2020). According to the WHO's current global report (Tramadol Update Review Report, 2014), dependence is low and usually occurs during long‐term use, while abuse is rarely a problem, so the risk of dependency can still be considered low compared with strong opioids. By contrast, the WHO Expert Committee on Drug Dependence has already considered scheduling tramadol as a narcotic drug under the Single Convention on Narcotic Drugs (Norris et al., 2021).

Regarding chronic non‐cancer pain, recent European recommendations state that opioids should only be used in certain indications (e.g. rheumatoid arthritis, osteoporosis) and under specified conditions (e.g. if other accepted non‐pharmacological and pharmacological therapies were not successful) (Häuser, Morlion, et al., 2021). In our study, indication‐linked reimbursement categories shed light on increasing opioid prescribing for musculoskeletal pain. The marked increase in fentanyl patches and oxycodone products in the high reimbursement category indicated that opioids were used in this indication more and more frequently. Similar trends of increased opioid use in non‐cancer pain were described in the United Kingdom between 2000 and 2010 (Zin et al., 2014) and in Germany between 1985 and 2016 (Rosner et al., 2019). Opioids play an important role in chronic rheumatologic conditions, particularly in the pain management of degenerative vertebral and large joints disorders. Their use is favourable when NSAIDs are contraindicated or not recommended. However, fear of addiction has hindered opioid use in rheumatology for several years in Hungary, and the approach to pain therapy has only changed recently. Increased opioid use certainly means that doctors are more vigilant about pain as a symptom, which is an encouraging trend.

In spite of the steadily rising number of new cancer cases in Hungary (Hungarian Central Statistical Office, n.d.), opioid utilization for cancer pain relief was surprisingly low and declined significantly during our study period. In Hungary, the annual number of deaths caused by cancer is approximately 32,000 (Hungarian Death Statistics, n.d.). That is around 0.3% of the population, which is similar to other European countries (Population Fact Sheets of the Global Cancer Observatory, 2020). Low opioid utilization in cancer pain might be explained by new and advanced targeted cancer treatment options that can decrease cancer pain by reducing tumour size. The combination of NSAIDs and adjuvants might also be a favourable alternative to opioid analgesia.

Hungary has strict prescribing regulations regarding strong opioids. Both specialists and general practitioners (GPs) can prescribe them but in long‐term therapy, the GP is the designated prescriber. Prescriptions must meet the statutory formal requirements, and with the exception of urgent dispensation, strong opioid prescriptions shall be accompanied by a formal notification from the prescribing GP every 3 month. Prescribers and pharmacies are legally obliged to keep a number of records of every activity regarding strong opioids. Although these administrative burdens are quite manageable compared with legislations in some other countries (Alqueres et al., 2015), they might discourage prescribers from opioid therapy. In case of weak opioids, there are no such legal criteria so tramadol and tramadol combination products might seem like an appealing alternative to strong opioids. This may be a plausible explanation for the high utilization rate of these products. However, a recent study evaluating the factors withholding prescribers from pain medication finds no correlation between regulatory barriers and access to strong opioids (Vranken et al., 2018), so other factors (e.g. fear of dependence and abuse, misunderstanding of these mechanisms) must also influence prescribing habits. Lack of clinical guidelines might also be a reason for low opioid utilization. In Hungary, only two clinical guidelines mentioned opioids, none of them in relation to cancer pain (one was about rheumatological diseases and the other about neuropathic pain; both of them expired within the study period).

The WHO views national opioid utilization as an indicator of progress in effective pain relief, particularly in regard to the treatment of cancer pain (Zin et al., 2014). A large increase in metrics does not equal that cancer patients are better supplied with opioids (Schubert et al., 2013) but low numbers might indicate underuse. Because Hungarian opioid utilization could be considered low compared with other countries, we presume opioid underuse in Hungary.

### 4.1 Strengths and limitations

A major strength of this study is that, as far as we know, this was the first national study describing the trends of opioid utilization in ambulatory care in Hungary. Our study is highly comprehensive because we used a large database that provided nationwide data covering almost 100% of opioid use. Moreover, our paper provides a detailed analysis of opioid utilization over a long period of time (15 years). Another strength is the use of three volume‐based metrics. In agreement with recent opioid utilization studies (Karanges et al., 2018; Nissen et al., 2019), we found that using multiple metrics enables comparison and helps to avoid misinterpretation of results and reduce bias that stem from the limitations of any given metric.

Our study has limitations. Firstly, our data sets did not contain individual‐level information (e.g. prescribed dose, duration of treatment) so we could not appraise the quality of prescribing. Our data also lacked information on hospital‐based treatments and on non‐pharmacological treatment options and the extent of their use in analgesia. Secondly, our national opioid utilization might be slightly underestimated because some tramadol combination drugs were not reimbursed, so there was no information on them in our database. However, the utilization of these products is presumably minor (because of their high price and lack of reimbursement), so it is unlikely that this had a significant effect on our findings.

No data were found on how to convert DDD to OME for rectal formulations so these were excluded from the OME‐related data.

Our study found an overall increase in opioid utilization between 2006 and 2020. However, we could conclude with certainty that there was no opioid epidemic in Hungary; moreover, our low utilization numbers might indicate underuse of opioid analgesia in ambulatory care.

## AUTHOR CONTRIBUTIONS

Conceptualisation: G.S., R.B., M.M., Z.E.; Data curation: M.M., Z.E.; Formal analysis: M.M., Z.E., R.B., R.V., P.D., D.C.; Methodology: G.S., M.M., Z.E., R.B., R.V., P.D., D.C.; Validation: G.S.; D.S., M.C., E.C., A.B.; Visualization: M.M., Z.E.; Writing—original draft preparation: Z.E., M.M., R.B.; writing—review and editing: all authors. All authors have read and agreed to the final version of the submitted manuscript.

## FUNDING INFORMATION

Project No. TKP2021‐EGA‐32 has been implemented with the support provided by the Ministry of Innovation and Technology of Hungary from the National Research, Development and Innovation Fund, financed under the TKP2021‐EGA funding scheme.

## CONFLICT OF INTEREST

The authors declare no conflict of interest.

## Supporting information


Table S1
Table S2
Figure S1
Figure S2
Figure S3
Figure S4
Figure S5
