# Supplementary material for: Trends in opioid utilization in Hungary, 2006–2020: A nationwide retrospective study with multiple metrics
Source: Eur J Pain. 2022 Aug 12;26(9):1896–909. doi: 10.1002/ejp.2011 (PMC9541344; doi:10.1002/ejp.2011)
Supplement: Supplementary file 1 — Table S1 Table S2 Figure S1 Figure S2 Figure S3 Figure S4 Figure S5 [file EJP-26-1896-s001.docx]

**Supplementary Material**

|  |  | **Reimbursement category** | | | |
| --- | --- | --- | --- | --- | --- |
| **ATC WHO** | **WHO Active Ingredient** | **General** | **High (musculoskeletal)** | **Accentuated (oncological)** | **Other** |
| **N02AA01** | morphine | x |  | x | x |
| **N02AA03** | hydromorphone | x |  | x | x |
| **N02AA05** | oxycodone | x | x ^a^ | x | x |
| **N02AA08** | dihydrocodeine | x |  | x | x |
| **N02AA55** | oxycodone and naloxone |  |  | x |  |
| **N02AA59** | codeine combinations excl. psycholeptics | x |  | x | x |
| **N02AB02** | pethidine | x |  | x |  |
| **N02AB03** | fentanyl | x | x ^b^ | x | x |
| **N02AE01** | buprenorphine | x |  | x | x |
| **N02AF02** | nalbuphine | x |  |  |  |
| **N02AJ06** | codeine and paracetamol | x |  |  |  |
| **N02AJ13** | tramadol and paracetamol | x |  | x | x |
| **N02AX02** | tramadol | x |  | x | x |

TableS1. Active opioid ingredients and their possible reimbursement categories (a: 5, 10 and 20 mg products; b: 12 and 25 µg/h patches)

| **ATC_WHO_** | **Active ingredient** | **Administration route** | **OME conversion factor** | **Reference** |
| --- | --- | --- | --- | --- |
| N02AA01 | morphine | parenteral | 3 | Nielsen et al., 2016 |
| N02AA01 | morphine | oral | 1 | Nielsen et al., 2016 |
| N02AA03 | hydromorphone | oral | 5 | Nielsen et al., 2016 |
| N02AA05 | oxycodone | oral | 1.5 | Nielsen et al., 2016 |
| N02AA08 | dihydrocodeine | oral | 0.1 | Nielsen et al., 2016 |
| N02AA55 | oxycodone and naloxone | oral | 1.5 | Nissen et al., 2019 |
| N02AJ06 | codeine and paracetamol | oral | 0.1 | Nielsen et al., 2016 |
| N02AA59 | codeine combinations excl. psycholeptics | rectal | no data in literature | |
| N02AB02 | pethidine | parenteral | 0.4 | Nielsen et al., 2016 |
| N02AB02 | pethidine | oral | 0.4 | Karanges et al., 2018 |
| N02AB03 | fentanyl | transdermal | 2.7 | Nielsen et al., 2016 |
| N02AE01 | buprenorphine | sublingual | 38.8 | Nielsen et al., 2016 |
| N02AE01 | buprenorphine | transdermal | 2.2 | Nielsen et al., 2016 |
| N02AF02 | nalbuphine | parenteral | 3 | Nielsen et al., 2016 |
| N02AJ13 | tramadol and paracetamol | oral | 0.2 | Nielsen et al., 2016 |
| N02AX02 | tramadol | parenteral | 0.24 | Karanges et al., 2018 |
| N02AX02 | tramadol | oral | 0.2 | Nielsen et al., 2016 |
| N02AX02 | tramadol | rectal | no data in literature | |

TableS2. OME conversion factors used for the calculation of OME per 1000 inhabitants per day

*OME/1000 inhabitants/day=DDD/1000 inhabitants/day × DDD_WHO_ × OME conversion factor**

** see in TableS2.*

FigureS1. Calculation of OME per 1000 inhabitants per day (based on the methodology of Karanges et al., 2018**)**

****FigureS2. Total opioid utilisation in the ambulatory sector in Hungary, 2006-2020

FigureS3. Utilisation of weak and strong opioids in the ambulatory sector in Hungary, 2006-2020

FigureS4. Opioid utilisation in the ambulatory sector in Hungary considering administration routes, 2006-2020

FigureS5. Opioid utilisation in the ambulatory sector in Hungary considering reimbursement categories, 2006-2020

**References**

Karanges, E. A., Buckley, N. A., Brett, J., Blanch, B., Litchfield, M., Degenhardt, L., & Pearson, S.-A. (2018). Trends in opioid utilisation in Australia, 2006-2015: Insights from multiple metrics. *Pharmacoepidemiology and Drug Safety*, *27*(5), 504–512. https://doi.org/10.1002/pds.4369

Nielsen, S., Degenhardt, L., Hoban, B., & Gisev, N. (2016). A synthesis of oral morphine equivalents (OME) for opioid utilisation studies: Oral Morphine Equivalents. *Pharmacoepidemiology and Drug Safety*, *25*(6), 733–737. https://doi.org/10.1002/pds.3945

Nissen, S. K., Pottegård, A., & Ryg, J. (2019). Trends of Opioid Utilisation in Denmark: A Nationwide Study. *Drugs - Real World Outcomes*, *6*(4), 155–164. https://doi.org/10.1007/s40801-019-00163-w
